# Supplementary material for: A Critical View of the Application of the APEX Software (Aqueous Photochemistry of Environmentally-Occurring Xenobiotics) to Predict Photoreaction Kinetics in Surface Freshwaters
Source: Molecules. 2019 Dec 18;25(1):9. doi: 10.3390/molecules25010009 (PMC7017383; doi:10.3390/molecules25010009)
Supplement: Supplementary file 1 [file molecules-25-00009-s001.zip › 3.2.4_gcc-4.4.0.zip!/3.2.4_gcc-4.4.0/3.2.4_gcc-4.4.0/doc/octave/html/liboctave/Contributors.html]

Contributors - Octave C++ Classes


Previous: Acknowledgements,
Up: Acknowledgements


---

### Contributors to Octave

In addition to John W. Eaton, several people have written parts
of liboctave. (This has been removed because it is the same as what is
in the Octave manual.)
